# Translation and validation of the functional assessment of cancer therapy-endometrial cancer (FACT-En) version 4 quality of life instrument into Thai language

**DOI:** 10.1186/s41687-026-01107-z

**Published:** 2026-06-03

**Authors:** Rachadapan Chaitosa, Arb-aroon Lertkhachonsuk, Bualuang Sumdarngrit

**Affiliations:** 1 Department of Obstetrics and Gynecology, Faculty of Medicine Ramathibodi Hospital, Mahidol University, Bangkok, Thailand; 2 Ramathibodi School of Nursing Faculty of Medicine, Ramathibodi Hospital, Mahidol University, Bangkok, Thailand

**Keywords:** FACT-En, Endometrial cancer, Quality of life, Thai version, Reliability, Validity

## Abstract

**Background:**

Quality-of-life assessment plays an essential role in evaluating outcomes and supportive care among women with endometrial cancer. This study aimed to translate, culturally adapt, and validate the Thai version of the *Functional Assessment of Cancer Therapy–Endometrial Cancer (FACT-En)* questionnaire.

**Methods:**

The English version of the FACT-En was translated and back-translated according to the FACIT translation methodology. A cross-sectional study was conducted among 173 Thai women with endometrial cancer at Ramathibodi Hospital, Mahidol University. Internal consistency was assessed using Cronbach’s alpha, and construct validity was evaluated using Spearman’s correlation with the WHOQOL-BREF-THAI.

**Results:**

The Thai FACT-En demonstrated excellent internal consistency, with Cronbach’s alpha coefficients ranging from 0.85 to 0.91 across subscales and 0.86 for the total score. Subscale scores showed moderate to strong correlations with the WHOQOL-BREF-THAI total score (*r* = 0.59–0.73, *p* < 0.01), supporting convergent validity.

**Conclusion:**

The Thai version of the FACT-En is a reliable and valid tool for assessing health-related quality of life in Thai women with endometrial cancer. It is suitable for use in both clinical practice and research to support patient-centered care and outcome evaluation.

## Introduction

Endometrial cancer is the most common gynecologic malignancy in developed countries and an increasing public health concern in Thailand. According to GLOBOCAN 2022, Thailand reported approximately 4,248 new cases and 1,301 deaths annually, making it the eighth most common cancer and tenth leading cause of cancer-related mortality among Thai women [1]. Early detection and treatment advances improve survival, but long-term physical, emotional, and social impacts highlight the need for integrating quality of life (QoL) assessment into routine care.

Patient-reported outcome measures (PROMs) are essential for evaluating treatment effectiveness beyond traditional clinical endpoints and for guiding patient-centered care. PROMs also provide critical evidence for health policy development, resource allocation, and decision-making within healthcare systems, ensuring that patient perspectives are incorporated into broader health system priorities. Disease-specific QoL instruments provide more precise insights than generic measures by addressing symptoms and concerns unique to the patient population.

The Functional Assessment of Cancer Therapy-Endometrial (FACT-En) consists of the 27-item FACT-G, which assesses four core quality-of-life domains—Physical Well-being, Social/Family Well-being, Emotional Well-being, and Functional Well-being—along with a 16-item Endometrial Cancer Subscale (EnCS) addressing disease-specific symptoms, such as fatigue, abdominal swelling, urinary frequency, and post-surgical sexual dysfunction [2–6]. Although the FACIT system lists the FACT-En as available in multiple languages including Chinese, French, German, Italian, Japanese, Korean, Russian, Spanish, and Turkish no peer-reviewed publication has reported cross-cultural adaptation or psychometric validation of the instrument in any language to date.

In Thailand, only the FACT-G has previously been translated and validated, whereas the EnCs had never undergone translation or formal evaluation. For this study, the version of the EnCs was newly translated by the primary author following the official FACIT translation methodology. Therefore, this study represents the first published translation, cultural adaptation, and psychometric validation of the complete FACT-En for Thai-speaking women with endometrial cancer, addressing a critical evidence gap and supporting its application in clinical practice, research, and health outcome measurement [7–8].

This study therefore aimed to translate and culturally adapt the FACT-En into Thai and to evaluate its psychometric properties, including internal consistency, construct validity, and convergent validity with WHOQOL-BREF-Thai. By establishing the validity and reliability of the Thai FACT-En, this work provides an essential tool for assessing QoL in Thai women with endometrial cancer, supporting both clinical decision-making and evidence-informed health policy within the Thai healthcare system.

## Materials and methods

### Study design and ethical considerations

This cross-sectional study was conducted at the Gynecologic Oncology Clinic, Ramathibodi Hospital, Mahidol University, between October 2019 and October 2022. Ethical approval was obtained from the Human Research Ethics Committee of Ramathibodi Hospital (Approval No. MURA2019/823). Written informed consent was obtained from all participants prior to enrollment.

### Participants

A total of 173 women with histologically confirmed endometrial cancer were recruited. Inclusion criteria included age ≥ 18 years, ability to communicate in Thai, and absence of cognitive impairment.

Although the FACT-En contains 43 items, factor analysis in this study focused exclusively on the 16-item Endometrial Cancer Subscale (EnCS). Based on common psychometric recommendations of 5–10 participants per item, the required sample size for the EnCS ranged from 80 to 160 participants. Therefore, the final sample of 173 participants met the recommended threshold and was considered adequate for evaluating reliability and construct validity of the Thai FACT-En.

### Participant characteristics

Participant sociodemographic and clinical characteristics are summarized in Table 1. The mean age was 59.96 years (SD = 11.35, range = 22–88). More than half (57.2%) had completed a college or university education or higher, and 41.0% were employed. Most participants (56.1%) underwent exploratory laparotomy, and 68.2% were diagnosed at an early stage according to the International Federation of Gynecology and Obstetrics (FIGO) 2009 staging system (stage I–II). A total of 63.01% received adjuvant treatment (chemotherapy and/or radiotherapy), while 36.98% received no adjuvant therapy.

**Table 1:** Presents the sociodemographic and clinical characteristics of the participants

Characteristics	Number of patients (N)	%
**Age (year)**		
Range	22-88	
Mean (SD)	59.96 (11.35)	
**Education**		
Less than high school	47	27.17
High school graduate	27	15.61
college/university or higher	99	57.23
**Occupation**		
Employed	71	41.04
Unemployed	102	58.96
**Type of Surgery**		
Explore laparotomy	97	56.07
Laparoscopic surgical staging	76	43.93
**Stage (FIGO 2009)**		
I – II	118	68.21
III – IV	39	22.54
N/A	16	9.25
**Treatment**		
Adjuvant treatment	109	63.01
No adjuvant treatment	64	36.98
**Total**	173	100.00

Note: Percentages may not total 100% due to rounding. FIGO = International Federation of Gynecology and Obstetrics, 2009 version

### Translation and cultural adaptation process [7, 8]

The validated Thai version of the FACT-G was used directly. Only the 16-item EnCS required translation and cultural adaptation following the FACIT translation methodology:

1. **Forward Translation**: Two independent bilingual translators translated the EnCS into Thai.
2. **Synthesis**: Translators and investigators reconciled discrepancies to produce a single synthesized version.
3. **Backward Translation**: Two native English speakers, blinded to the original, independently back-translated the synthesized Thai version.
4. **Expert Committee Review**: A multidisciplinary panel reviewed all versions to ensure semantic, idiomatic, and conceptual equivalence and to incorporate cultural adaptations where needed.
5. **Pilot Testing**: The Thai EnCS was pilot-tested with 10 patients to assess clarity, acceptablility, and cultural relevance. No further modifications were required.

### Instruments

- **FACT-En** [2, 3]: 43-item instrument comprising FACT-G (27 items) and EnCS (16 items). Items are scored on a 5-point Likert scale (0 = Not at all to 4 = Very much), with higher scores indicating better quality of life. Subscales include Physical Well-being (PWB), Social/Family Well-being (SWB), Emotional Well-being (EWB), Functional Well-being (FWB), and EnCS. The Trial Outcome Index (TOI) sums PWB, FWB, and EnCS scores.
- **WHOQOL-BREF (Thai version)** [9, 10]: 26-item generic QoL measure covering four domains Physical, Psychological, Social, and Environmental. Items are rated on a 5-point Likert scale, with higher scores indicate better QoL. The total score ranges from 26 to 130.

### Data collection

Trained research assistants administered the questionnaires via face-to-face interviews. Clinical and demographic data were extracted from medical records.

### Statistical analysis

All analyses were performed using SPSS version 15.0. Descriptive statistics were used to summarize participant characteristics and item responses. Missing data were handled using available-case analysis, no imputation was performed. The number and percentage of missing responses for each EnCS item are reported in Table 2.

**Table 2:** Item-level response distribution for the 16 Endometrial Cancer Subscale (EnCS) items

Symptom	Not at all	A little bit	Somewhat	Quite a bit	Very much	Missing	% Missing
Stomach swelling	80	56	20	15	1	1	0.6%
Stomach cramps	68	57	30	15	3	0	0.0%
Stomach pain or discomfort	49	77	26	19	2	0	0.0%
Vaginal bleeding	140	28	2	3	0	0	0.0%
Vaginal discharge	140	23	5	3	1	1	0.6%
Change in appearance	125	26	13	8	1	0	0.0%
Hot flushes	116	28	19	7	2	1	0.6%
Cold sweats	115	31	19	4	1	3	1.7%
Night sweats	112	42	10	7	1	1	0.6%
fatigue	74	62	22	14	1	0	0.0%
Pain during intercourse	103	8	1	1	0	60	34.7%
Difficulty digesting food	53	54	36	23	6	1	0.6%
Shortness of breath	97	42	19	11	3	1	0.6%
Constipation	64	52	30	20	7	0	0.0%
Urinate frequency	60	34	37	38	4	0	0.0%
Pelvic pain or discomfort	81	56	24	11	1	0	0.0%

Note. This table shows the distribution of responses and missing data for the 16 EnCS items. The highest missing rate occurred for the item on pain or discomfort with intercourse (34.7%), reflecting the sensitivity of this question

### Internal consistency reliability

Cronbach’s alpha coefficients were calculated for each subscale and the total scores, with α ≥ 0.70 indicating acceptable reliability.

### Construct validity

#### Exploratory factor analysis (EFA)

Construct validity was assessed using EFA of the 16-item EnCS only. Principal component analysis with varimax rotation was applied. Factors were extracted using the criterion of eigenvalues > 1, supported by visual inspection of the scree plot. Items with factor loadings ≥ 0.40 were considered to load meaningfully on a factor.

### Convergent and divergent validity

Spearman’s rank correlations were then computed between the Thai FACT-En subscale / total scores and WHOQOL-BREF-Thai domain / total scores. Strong correlations between conceptually related domains supported convergent validity, weak correlations supported divergent validity.

### Group comparisons

Independent samples t-tests compared QoL and symptom scores between patients receiving adjuvant therapy (*n* = 109) and those not receiving adjuvant therapy (*n* = 64).

## Results

### Participant flow

**Fig. 1:**
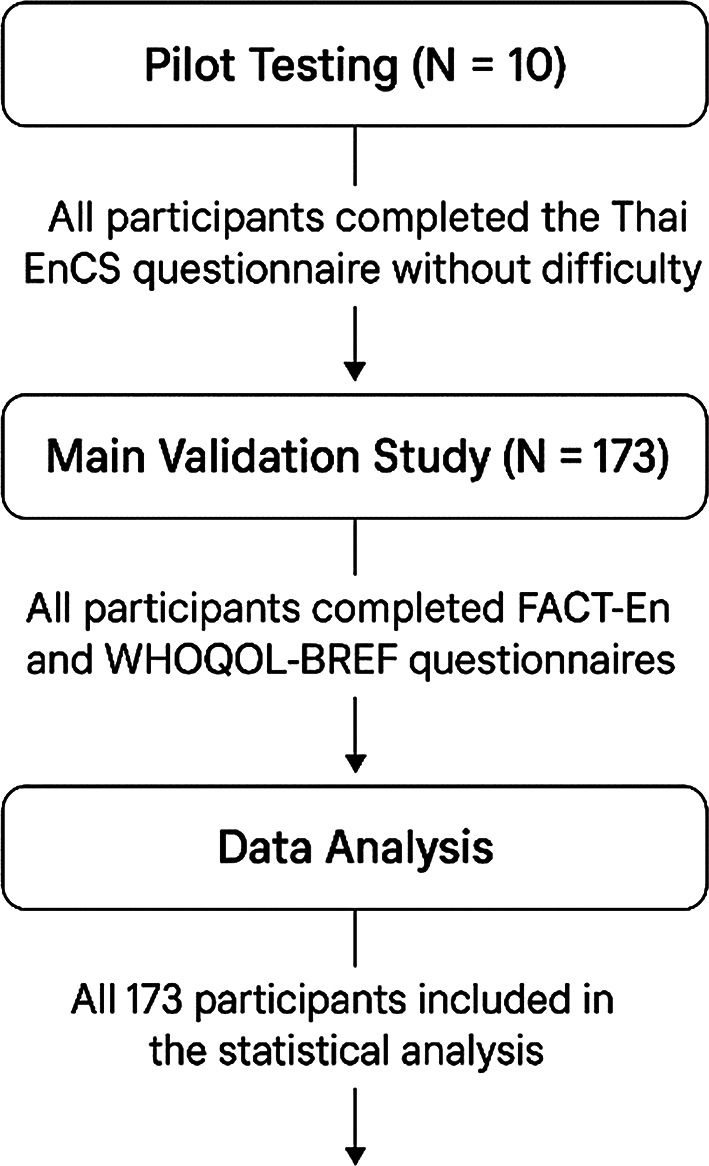
Flowchart: Participant enrollment and study process. Flow of participants through the study. Ten endometrial cancer patients participated in the pilot testing of the Thai EnCS. The main validation study included 173 participants, all of whom completed the FACT-En and WHOQOL-BREF questionnaires and were included in the statistical analysis

Figure 1 illustrates the flow of participants through the study. Ten endometrial cancer patients participated in the pilot testing of the Thai Endometrial Cancer Subscale EnCS, all of whom completed the questionnaire without difficulty. The main validation study included 173 participants, all of whom completed the FACT-En and WHOQOL-BREF questionnaires and were included in the statistical analysis.

### Pilot test of EnCS

The pilot testing of the Thai EnCS was conducted with 10 endometrial cancer patients, all of whom were of the same patient type as those in the main validation study, to assess item clarity, acceptability, and cultural appropriateness. All participants completed the questionnaire without difficulty, and no revisions were required. Mean item scores ranged from 0.10 to 1.30 (SD = 0.32–1.39), reflecting a low to moderate symptom burden among respondents. The Cronbach’s alpha coefficients if an item was deleted ranged from 0.83 to 0.88, and the overall Cronbach’s alpha for the scale was 0.86, indicating good internal consistency reliability. These findings confirmed that the translated EnCS items were linguistically clear, culturally relevant, and psychometrically sound for use in the main validation study (Table 3).

**Table 3:** Pilot testing of the Thai FACT-En Endometrial Cancer Subscale (EnCS) (*n* = 10)

Item	Mean	SD	Cronbach’s α if item deleted
Stomach sweeling	0.80	1.39	0.84
Stomach cramps	1.00	1.33	0.83
Stomach pain or discomfort	0.70	1.34	0.84
Vaginal bleeding	0.10	0.32	0.88
Vaginal discharge	0.10	0.32	0.88
Chang in apperarance	0.70	0.82	0.85
Hot flushes	0.40	0.69	0.87
Cold sweats	0.40	0.67	0.88
Night sweats	0.30	0.68	0.88
Fatigue	1.00	1.33	0.84
Pain during intercourse	0.40	1.27	0.84
Difficulty digesting food	1.00	1.25	0.84
Shortness of breath	0.70	1.34	0.84
Constipation	1.00	1.25	0.87
Urinate frequency	1.30	0.95	0.88
Pelvic pain or discomfort	0.50	0.71	0.86
**Overall Cronbach’s α**			**0.86**

Note: The Thai version of the Endometrial Cancer Subscale (EnCS) was pilot-tested with 10 endometrial cancer patients to evaluate clarity, acceptability, and cultural relevance. Mean item scores ranged from 0.10 to 1.30, indicating low to moderate symptom burden. The overall Cronbach’s alpha coefficient (α = 0.86) demonstrated good internal consistency reliability

**Symptom Profile of Participants (Descriptive Item Responses)** (Table 2) presents the distribution of responses for each EnCS symptom item. Most participants reported low to moderate symptom severity across gastointestinal, vasomotor, appearance-related, and pelvic symptoms. The highest proportion of missing data was observed for the item on pain or discomfort during intercourse (34.7%), which is consistent with previous studies reporting limited sexual activity following treatment for endometrial cancer.

### Internal consistency of Thai FACT-En

The Thai FACT-En exhibited strong internal consistency reliability, with Cronbach’s alpha ranging from 0.85 to 0.91 across subscales. The total scale score demonstrated good reliabibility (Cronbach’s alpha = 0.86), supporting the stability of the instrument (Table 4).

**Table 4:** Descriptive statistics and internal consistency reliability of the FACT-En subscale and total scores (*N* = 173)

Subscale / Total score	Number of items	Mean ± SD	Range	Cronbach’s α
Physical Well-Being (PWB)	7	21.16 ± 5.24	0–28	0.89
Social/Family Well-Being (SWB)	7	21.40 ± 4.52	0–28	0.91
Emotional Well-Being (EWB)	6	18.23 ± 4.86	0–24	0.89
Functional Well-Being (FWB)	7	17.23 ± 6.09	0–28	0.88
Endometrial Cancer Subscale (EnCS)	16	51.65 ± 9.20	0–64	0.88
Trial Outcome Index (TOI) †	30	89.97 ± 17.05	0-120	0.85
FACT-G Total Score	27	77.97 ± 15.44	0–108	0.85
FACT-En Total Score	43	129.95 ± 22.42	0–160	0.86

Note: Higher scores indicate better quality of life

†TOI = sum of PWB + FWB + EnCS subscales

Cronbach’s α ≥ 0.70 indicates acceptable internal consistency reliability

### Exploratory factor analysis (EFA)

The EFA of the 16-item EnCS produced a multi-factor solution consistent with clinically meaningful symptom clusters. Vasomotor items (cold sweats, hot flashes, night sweats) loaded strongly onto a single factor, while gastrointestinal and pelvic symptoms (constipation, digestive problems) also showed strong loadings on a separate factor. Sexual symptoms and appearance concerns demonstrated a distinct factor pattern, and vaginal discharge formed a separate high-loading factor. The rotated factor loadings are presented in Table 5.

**Table 5:** Factor loadings for the Thai FACT-En (EFA, Varimax rotation)

Item	PWB	FWB	EWB	SWB	EnCS
Shortness of breath	0.726	0.273	0.272	0.032	0.007
Fatigue	0.708	0.176	0.185	0.013	0.198
Pelvic pain or discomfort	0.657	0.137	-0.095	0.339	-0.178
Stomach pain or discomfort	0.597	0.098	0.244	0.521	-0.241
Urinary frequency	0.516	0.140	0.242	0.022	0.100
Pain during intercourse	0.460	-0.121	-0.347	0.083	0.424
Change in appearance	0.438	0.276	0.228	0.030	-0.017
Cold sweats	0.215	0.908	0.021	0.137	0.048
Hot flashes	0.193	0.857	0.136	0.005	-0.045
Night sweats	0.211	0.799	0.140	0.237	0.090
Constipation	0.193	0.079	0.833	0.077	0.105
Difficulty digesting food	0.463	0.147	0.721	0.045	0.057
Vaginal bleeding	-0.010	0.059	-0.197	0.767	0.304
Stomach swelling	0.142	0.210	0.354	0.731	0.147
Stomach cramps	0.219	0.242	0.477	0.490	-0.305
Vaginal discharge	0.035	0.106	0.170	0.191	0.838

Note: PWB = Physical Well-Being; SWB = Social/Family Well-Being; EWB = Emotional Well-Being; FWB = Functional Well-Being; EnCS = Endometrial Cancer Subscale. These abbreviations represent the FACT-En subscales

### Construct, convergent, and divergent validity

The Thai FACT-En showed strong positive correlations with the WHOQOL-BREF-Thai total score (rₛ = 0.59–0.73, *p* < 0.01). Subscale-level analysis demonstrated the expected pattern of convergent validity: PWB correlating most strongly with the Physical domain (rₛ = 0.68), and EWB correlating most strongly with the Psychological domain (rₛ = 0.71). These findings support the construct validity of the Thai FACT-En (Table 6).

**Table 6:** Spearman’s correlation coefficients between the Thai FACT-En and WHOQOL-BREF-Thai domains (*N* = 173)

Thai FACT-En domains	Physical domain	Psychological domain	Social domain	Environmental domain	Total WHOQOL-BREF
Physical Well-Being (PWB)	0.68**	0.55**	0.41**	0.49**	0.62**
Social/Family Well-Being (SWB)	0.39**	0.46**	0.64**	0.48**	0.59**
Emotional Well-Being (EWB)	0.48**	0.71**	0.43**	0.55**	0.66**
Functional Well-Being (FWB)	0.64**	0.60**	0.45**	0.50**	0.68**
Endometrial Cancer Subscale (EnCS)	0.62**	0.51**	0.40**	0.47**	0.60**
Trial Outcome Index (TOI) †	0.72**	0.63**	0.49**	0.54**	0.70**
FACT-En Total	0.69**	0.70**	0.56**	0.58**	0.73**

Note: All correlation coefficients were significant at p < 0.01

### Group comparisons

Independent samples t-tests comparing patients who received adjuvant therapy with those who did not showed no statistically significant differences in total FACT-En, subscale, or Trial Outcome Index (TOI) scores (all *p* > 0.05), indicating no detectable association between adjuvant treatment and overall quality of life in this sample (Table 7).

**Table 7:** Comparison of Thai FACT-En scores between adjuvant therapy and no adjuvant therapy groups (*N* = 173)

Domain / Subscale	Adjuvant therapy (*n* = 109) Mean ± SD	No adjuvant therapy (*n* = 64) Mean ± SD	t-value	*p*-value
Physical Well-being (PWB)	20.64 ± 6.24	22.19 ± 4.86	-1.89	0.061
Social/Family Well-being (SWB)	21.13 ± 4.44	21.95 ± 4.68	-1.37	0.173
Emotional Well-being (EWB)	18.23 ± 4.90	18.21 ± 4.82	0.03	0.977
Functional Well-being (FWB)	16.80 ± 6.24	18.09 ± 5.75	-1.23	0.221
Endometrial Cancer Subscale (EnCS)	52.00 ± 8.54	50.97 ± 10.43	0.68	0.497
Trial Outcome Index (*TOI)	89.33 ± 17.21	91.22 ± 16.82	-0.64	0.524
FACT-G Total	76.72 ± 15.81	80.43 ± 14.51	-1.41	0.161
FACT-En Total	129.21 ± 23.21	131.40 ± 20.88	-0.77	0.442

Note: Independent samples t-tests were used to compare mean scores between groups

All comparisons were non-significant (p > 0.05)

*TOI = Trial Outcome Index (sum of PWB, FWB, and ECS scores)

## Discussion

This study represents the first culturally adapted and validated Thai version of the FACT-En, combining the previously published FACIT Thai FACT-G with a newly translated EnCS. The instrument demonstrated good internal consistency and acceptable construct validity among Thai women with endometrial cancer in a single-center setting.

### Reliability

Internal consistency was highest for the SWB subscale (α = 0.91), reflecting strong coherence among items and the cultural importance of family support. The FWB subscale showed lower mean scores, suggesting functional challenges following diagnosis and surgery, consistent with findings from previous studies in other settings. Although FWB demonstrated slightly lower internal consistency (α = 0.88), all subscales remained within acceptable ranges [11, 12].

### Construct validity

High correlations between the Thai FACT-En and the WHOQOL-BREF-Thai total score support convergent validity [13]. Moderate to high correlations were observed for SWB and FWB (0.59 and 0.68, respectively), indicating that these subscales relate positively to overall quality of life. No evidence of divergent validity was observed, consistent with cultural expectations of stable family support despite physical or functional limitations.

### Symptom assessment

The EnCS captured common symptoms such as fatigue, abdominal discomfort, and urinary frequency [14]. Missing data were minimal for nearly all items (0-1.7%), except for the item assessing discomfort or pain during intercourse, which showed a high rate of missing responses (34.7%). This pattern aligns with cultural norms in Thailand, where many women abstain from sexual activity following cancer diagnosis or surgery, contributing to non-response for partner-specific sexual items [15]. Similar findings have been reported in other conservative populations.

### Group comparisons

No significant differences were found between patients who received adjuvant therapy and those who did not, suggesting no detectable association between adjuvant therapy and quality of life in this sample. However, causal inferences cannot be drawn due to the cross-sectional design and moderate sample size.

#### Clinical implications

The Thai FACT-En is suitable for routine clinical use to assess patient-reported quality of life, guide individualized patient-centered care, and monitor symptom burden over time. It may also be used in health economic research, including estimating health utility values from disease-specific quality of life measures for QALY calculations and cost-effectiveness evaluations, facilitating the integration of patient perspectives into clinical and policy decision-making.

#### Limitations

This study was conducted at a single center with a moderate sample size, which may limit the generalizability of the findings. Some sensitive items, particularly the question on pain or discomfort during intercourse, had a high rate of missing responses, potentially affecting the completeness of the data. Additionally, the current sample size may be insufficient for robust factor analysis. Future research should include larger, multicenter populations and employ confirmatory factor analysis to further validate the Thai FACT-En.

## Conclusion

The Thai version of the Functional Assessment of Cancer Therapy–Endometrial (FACT-En) demonstrated good reliability, validity, and cultural appropriateness for assessing the quality of life in Thai-speaking women with endometrial cancer. By capturing both general and disease-specific aspects of well-being, the Thai FACT-En serves as a comprehensive and culturally sensitive tool for use in clinical practice, research, and health policy development. Its application can enhance patient-centered care, support outcome evaluation, and contribute to health economic analyses, including the estimation of health utility values for quality-adjusted life year (QALY) calculations within the Thai healthcare context.

## Data Availability

The datasets used and/or analyzed during the current study are available from the corresponding author upon reasonable request.
